# Disease Severity and Response to Induction Therapy in Hispanic Patients With Antineutrophilic Cytoplasmic Autoantibody-Associated Vasculitis-Related Diffuse Alveolar Hemorrhage

**DOI:** 10.7759/cureus.24470

**Published:** 2022-04-25

**Authors:** Christine E Loftis, Emilia C Dulgheru, Rosa White

**Affiliations:** 1 Internal Medicine, University of Texas Rio Grande Valley School of Medicine, Edinburg, USA; 2 Internal Medicine, Doctors Hospital at Renaissance, McAllen, USA; 3 Rheumatology, Doctors Hospital at Renaissance, McAllen, USA

**Keywords:** drug-induced vasculitis, hydralazine, cyclophosphamide, rituximab, plasmapheresis, diffuse alveolar hemorrhage, microscopic polyangiitis, granulomatosis with polyangiitis, anca vasculitis

## Abstract

Objectives

We examined the response to induction therapy of Hispanic patients with antibody-associated vasculitis (AAV)-related diffuse alveolar hemorrhage (DAH). This study aimed to determine the severity of disease at presentation and the response to induction therapy in our patient population.

Methods

We retrospectively reviewed the clinical data of Hispanic patients hospitalized with antineutrophil cytoplasmic antibody (ANCA) vasculitis between October 1, 2010, and December 31, 2021. We identified 98 Hispanic patients hospitalized with AAV and 19 admitted with AAV-related DAH. The Birmingham Vasculitis Activity Score (BVAS) was obtained from all patients on presentation.

Results

Based on the 2012 Revised International Chapel Hill Consensus Conference Nomenclature of Vasculitides, 12 patients met the diagnostic criteria for microscopic polyangiitis (MPA) and seven met the criteria for diagnosing granulomatosis with polyangiitis (GPA). All patients received methylprednisolone therapy. Induction therapy consisted of cyclophosphamide pulse therapy (n=3), cyclophosphamide plus plasmapheresis (PLEX) (n=1), rituximab induction therapy (n=8), and rituximab induction plus plasmapheresis (n=6), and one patient received one dose of cyclophosphamide followed by rituximab plus plasmapheresis. The average BVAS was 25.53 at presentation. Survival at six months included 67% (n=2) treated with cyclophosphamide alone, 75% (n=6) treated with rituximab alone, and 50% (n=3) treated with rituximab plus PLEX. The patient who received an initial loading dose of cyclophosphamide followed by rituximab plus PLEX did survive for six months; however, the patient treated with cyclophosphamide plus PLEX did not have early survival.

Conclusions

Hispanic patients with ANCA-associated vasculitis present with a more severe disease burden at presentation based on BVAS. Approximately 37% of our patient population had early death (death at <6 months) despite adhering to the standard of care for induction therapy. Due to the more significant disease burden at presentation, it is vital to include ethnic minorities in large clinical trials to help improve outcomes in these patient populations.

## Introduction

Antineutrophil cytoplasmic antibody (ANCA)-associated vasculitis (AAV) is a group of diseases characterized by inflammation and damage of small vessels within various organs with a predilection for the kidneys and upper and lower respiratory tract. One of the most feared presentations of AAV is diffuse alveolar hemorrhage (DAH), marked by hemoptysis, anemia, diffuse alveolar infiltrates on imaging, and hypoxemic respiratory failure. Over the past decade, several trials have been performed to determine the best therapeutic strategies for remission induction in patients with severe, active AAV. Despite advancements in therapy, mortality related to DAH remains high, with rates as high as 50%-65% depending on the study [1,2]. Few studies have discussed the disease burden, response to therapy, or mortality rate of AAV-related DAH within Hispanic populations. In 2014, Sreih et al. demonstrated that compared to Caucasian patients within the exact geographic location, Hispanic patients have a higher Birmingham Vasculitis Activity Score (BVAS) at presentation, more severe renal involvement, and overall worse disease burden [3]. Although their study did demonstrate more severe renal involvement among Hispanic patients, they did not compare outcomes of diffuse alveolar hemorrhage between groups. Furthermore, a study performed by Singh et al. concluded that Hispanic patients with AAV-related DAH had a similar response to induction therapy as the general population [4]. Given the scarcity of studies on Hispanic patients, we designed a retrospective study to assess disease severity at presentation and response to induction therapy of Hispanic patients diagnosed with AAV-related DAH in our community hospital.

## Materials and methods

Study design

We examined the response to induction therapy of Hispanic patients with AAV-related DAH. This study aimed to determine the severity of disease at presentation and the response to induction therapy in our patient population.

Inclusion and exclusion criteria

We filtered chart data by including the following ICD-10 codes: M31.30/M31.31, M30.1, M31.7, and M31.8. We included patients between the ages of 18 and 99, self-identified as Hispanic, hypoxemic based on the requirement of supplementation oxygen or mechanical ventilation, and presented with or had hemoptysis during hospitalization. Patients who did not self-identify as Hispanic were not included in the study.

Data collection

We identified a total of 98 Hispanic patients hospitalized with AAV. Of the 98 patients, 19 were admitted with AAV complicated by DAH between October 1, 2010, and December 31, 2021. Patients were analyzed based on age, gender, length of hospital stay, clinical manifestations, serology, organ involvement, radiographic findings, pathology reports, treatment modalities, outcomes, and survival at six months. In addition, the Birmingham Vasculitis Activity Score (BVAS) was obtained from all patients. Our study was approved by the Doctors Hospital at Renaissance Health Institute for Research and Development Institutional Review Board of Committee with the following IRBNet ID: 1742470-2.

## Results

**Figure 1:**
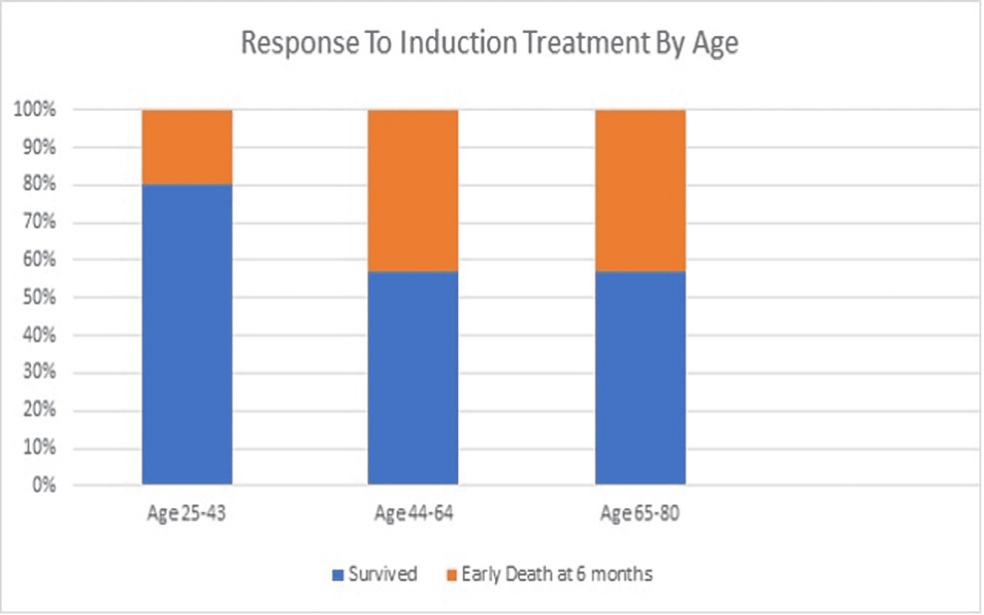
Post-induction treatment survival by age

We identified 19 Hispanic patients with AAV-related DAH. Of the 19 patients, six were males, and 13 were females. The mean age at presentation was 55 ± 18 years (Figure 1). Serology showed more patients with P-ANCA hospitalized with DAH than patients with C-ANCA pattern (seven patients with C-ANCA and 12 patients with P-ANCA pattern by immunofluorescence). In patients with available ELISA, anti-proteinase-3 antibodies were positive in four patients, and eight patients were positive for anti-myeloperoxidase antibodies. Based on the 2012 Revised International Chapel Hill Consensus Conference Nomenclature of Vasculitides, 12 patients met the diagnostic criteria for microscopic polyangiitis (MPA), and seven met the criteria for diagnosing granulomatosis with polyangiitis (GPA) [5].

Of the total patients, 84% (n=16) had a presentation of gross hemoptysis, while the remaining 16% (n=3) presented with dyspnea and later developed hemoptysis. Other concomitant symptoms included three patients with palpable purpura at presentation, one patient with keratitis, one patient with blurred vision, two patients with ischemic chest pain, 12 patients with myalgias or arthralgias, and three patients who also endorsed hematochezia. Furthermore, approximately one-third (n=6) of the patients were on hydralazine prior to admission; however, only one patient had an anti-histone antibody ordered and confirmed positive during evaluation. Of the patients on hydralazine, 83% (n=5) were P-ANCA-positive. Respiratory failure requiring mechanical ventilation occurred in 47% (n=9) of the patients. At presentation, 95% (n=18) of the patients had renal involvement, with half of those patients requiring hemodialysis (n=9), and 58% (n=11) had biopsy-proven pauci-immune-type necrotizing crescentic glomerulonephritis. A urine drug screen was positive for cocaine in one patient at initial diagnosis. Acute blood loss anemia, hypoxemia, and chest imaging showing diffuse bilateral infiltrate were present in all the patients. At presentation, the average BVAS (of new-onset/worsening of symptoms) was 25.53 (Table 1).

**Table 1:** Patient demographics, severity by BVAS, treatment, and outcomes F: female, M: male, PLEX: plasmapheresis

Age	Gender	Length of hospital stay	Serology	Treatment	Outcome	BVAS
67	M	10	P-ANCA	Rituximab/plasmapheresis	Survived	22
63	M	11	C-ANCA	Cyclophosphamide	Expired	39
79	F	15	P-ANCA	Rituximab/PLEX	Expired	11
32	F	9	C-ANCA	Rituximab	Expired	36
34	M	14	C-ANCA	Cyclophosphamide	Survived	19
57	M	14	C-ANCA	Rituximab	Expired	24
69	F	34	C-ANCA	Cyclophosphamide/PLEX	Expired	28
65	F	4	P-ANCA	Rituximab/PLEX	Survived	38
23	F	19	P-ANCA	Cyclophosphamide	Survived	21
70	M	39	P-ANCA	Rituximab/PLEX	Expired	22
78	F	13	P-ANCA	Rituximab	Survived	32
28	F	18	P-ANCA	Rituximab/PLEX/cyclophosphamide	Survived	27
56	F	8	P-ANCA	Rituximab	Survived	25
33	F	22	P-ANCA	Rituximab	Survived	18
50	F	16	P-ANCA	Rituximab	Survived	27
64	M	16	C-ANCA	Rituximab	Survived	32
54	F	21	P-ANCA	Rituximab/PLEX	Expired	19
56	F	19	C-ANCA	Rituximab	Survived	27
75	F	20	P-ANCA	Rituximab/PLEX	Survived	18

Regarding treatment, 95% (n=18) of the patients received high-dose pulse methylprednisolone, apart from one patient who received methylprednisolone at a lower dose of 40 mg every eight hours for five days. Induction therapy consisted of cyclophosphamide pulse therapy (n=3), cyclophosphamide plus plasmapheresis (n=1), rituximab induction therapy (n=8), and rituximab induction plus plasmapheresis (n=6), and one patient received one dose of cyclophosphamide followed by rituximab plus plasmapheresis. The average number of plasmapheresis sessions was 5.375.

Of the three patients treated with cyclophosphamide alone, 67% (n=2) survived to six months, with the average hospital stay being 14.67 days. The patient who received cyclophosphamide plus plasmapheresis did not survive for six months. She did survive her initial hospitalization; however, she presented one month afterward with similar symptoms resulting in a month-long hospital stay, which ultimately resulted in her demise. Of the patients treated with rituximab alone, 75% (n=6) had survived at six months. The average length of hospital stay for these patients was 14.625 days. Of the patients who received the combination of plasmapheresis with rituximab, 50% (n=3) had survived at six months. The average time of hospital stay was 18.17 days. Lastly, the patient treated with an initial loading dose of cyclophosphamide plus the combination of rituximab and plasmapheresis had survived six months. When survival at six months was analyzed by age group, 80% (n=4) of patients aged 25-43 survived, 57% (n=4) of patients aged 44-64 survived, and 57% (n=4) of patients aged 65-80 survived. Of the total patients in the study, 63% of the patients survived for six months (Figure 1).

## Discussion

Despite the life-threatening aspect of DAH in AAV, its treatment is not different from the management of AAV in general. Since the 1970s, the standard of care for AAV treatment has been oral cyclophosphamide [6]. Initially, treatment was daily dosing of cyclophosphamide; however, the option to use pulse cyclophosphamide was made available because of the findings of the CYCLOPS trial [7]. The only treatment for induction therapy of AAV for 40 years was cyclophosphamide. This changed in 2010 when Stone et al. published the RAVE trial in the New England Journal of Medicine (NEJM). The RAVE trial was a non-inferiority trial that compared rituximab at a dose of 375 mg per meter squared versus daily cyclophosphamide in the induction of remission of AAV [8]. The study results showed that rituximab therapy was not inferior to daily cyclophosphamide for the induction of remission in severe AAV while also showing to be superior in preventing relapsing disease. Because of this trial, the 2016 updated management guidelines for AAV options include either rituximab or cyclophosphamide in the induction of remission of AAV [9]. Despite the advances that were being made in the realm of AAV, there remained a concern that the treatment options were not always efficacious, the onset of action was delayed, and toxicity was vast; therefore, in 2020, the Cambridge University Hospitals NHS Foundation performed the PEXIVAS trial, which evaluated the use of plasma exchange in the treatment of severe, active AAV [10]. The study results showed that the use of plasmapheresis did not reduce the incidence of death or end-stage kidney disease. In 2021, the updated American College of Rheumatology/Vasculitis Foundation Guideline includes that rituximab is conditionally recommended over cyclophosphamide, and recommendations are against the addition of plasma exchange for the induction of remission. Guidelines also recommend that patients receive concomitant glucocorticoid treatment with either pulse methylprednisolone 500-1000 mg for 3-5 days or oral prednisone 1 mg/kg/day [11].

Historically, clinical trials have failed to enroll patients of diverse ethnic backgrounds. One of the most significant trials we have in AAV, RAVE, had nine Hispanic patients enrolled in the study [8]. Several small studies were published over the past decade to look at disease severity, mortality rates, and response to induction therapy among Hispanic patients. Sreih et al. performed a study to compare the severity and outcomes of AAV between 23 Hispanic patients and 32 Caucasian patients over six years in Chicago [3]. Their Hispanic cohort consisted of 69.6% (n=16) and 17.4% (n=4) of patients diagnosed with GPA and MPA, respectively. Unlike our study, they also included patients diagnosed with eosinophilic granulomatosis with polyangiitis (eGPA) and limited renal vasculitis. When comparing the two groups, they found that their Hispanic patients had a higher BVAS than Caucasians at presentation, with average scores of 16.3 versus 10.7 [3]. Furthermore, they also noted that 85% (n=20) of their Hispanic patients had renal involvement at presentation. Although we did not compare Hispanic and Caucasian patients in our study, we did note an average BVAS of 25.53 at the initial presentation. Our study specifically looked at AAV-related DAH and that, at presentation, these patients would likely have a higher BVAS than patients who were only being treated for severe, active disease without DAH. Like their study, 95% of our patients had renal involvement at presentation. This has been attributed to lower socioeconomic status in Hispanic patients and delays in presentation and treatment of early AAV [3].

Singh et al. performed a study looking at the response to induction therapy of AAV-related DAH in Hispanic patients in a large academic hospital in Los Angeles. The study included 13 Hispanic patients diagnosed with AAV-related DAH, and although the sample size was small, it was the first of its kind to investigate the response to induction therapy in a predominately Hispanic population with severe active disease [4]. Their cohort consisted of six men and seven women, whereas we included 13 women and six men. Nine were diagnosed with MPA, and four patients were diagnosed with GPA compared to 12 patients with MPA and seven with GPA in our study. As previous studies have mentioned, Hispanics from Latin America have a higher incidence of MPA, and our study also supports their findings [8]. Their treatment regimens consisted of cyclophosphamide (n=6), rituximab (n=4), cyclophosphamide plus rituximab (n=1), plasmapheresis (n=9), and IV gamma globulin (n=5). It was not clear which of their patients received plasmapheresis in addition to cyclophosphamide or rituximab. They concluded that their average BVAS was 21.2 ± 5.1 at presentation. We noted a slightly higher BVAS in our patient population at presentation. Of the 13 patients included in the study, 92% (n=12) had early survival at six months; however, only 63% (n=12) of our patients survived to six months. One could hypothesize that we had worse outcomes due to higher BVAS at presentation; however, higher BVAS did not correlate to worse outcomes based on our results. It is important to note that almost one-third of our patients were on hydralazine prior to the diagnosis of AAV, and it has been well documented in the literature that hydralazine can induce AAV and DAH [12]. Sandy Lee et al. studied a cohort of Hispanic patients in Southern California and concluded that clinicians treating Hispanic patients with ANCA-associated vasculitis should have a high index of suspicion for severe disease in this patient population. They also suggested that epidemiologic and disparity research should be conducted to evaluate the discrepancy in outcomes in these groups. Hispanics in this population were more likely to be admitted to the ICU, have more flares, reach end-stage renal disease, have severe pulmonary manifestations, and have fewer outpatient follow-up visits than their Caucasian counterparts [13].

One of the limitations of our study is the small study size. Therefore, although ANCA-related DAH is rare, we could not draw any conclusions due to too few subjects in the study. In addition, because we serve a large Hispanic population, there was a scarcity of patients who were non-Hispanic Whites that could be used to compare outcomes in our hospital, and thus, our results had to be based on a comparison of previously published articles.

## Conclusions

The results of our study align with other studies that have been performed in predominately Hispanic populations. Our results conclude that Hispanic patients present with a more severe disease burden at presentation and appear to have poor outcomes overall, with early death in 37% of our patient population despite adhering to a standard of care. We postulate that our patients have a more severe disease at presentation due to underlying chronic health issues, limited access to healthcare and health insurance, and poor health literacy. Of note, 63% (n=12) of our cohort were obese, 68% (n=13) had hypertension, 21% (n=4) had a preexisting lung condition, 21% (n=4) had cardiovascular disease, and 32% (n=6) had a diagnosis of diabetes mellitus. These factors likely contribute to delay in seeking care, which would postpone treatment intervention, resulting in worse outcomes. Although our sample size was small, our study highlights the importance of including ethnic minorities in more extensive prospective studies and clinical trials to assess their response to treatment and contribute to the development of treatment strategies that will improve outcomes.
